# Parent, child, and family outcomes following Acceptance And Commitment Therapy for parents of autistic children: A randomized controlled trial

**DOI:** 10.1177/13623613231172241

**Published:** 2023-05-11

**Authors:** Andrea L Maughan, Yona Lunsky, Johanna Lake, Jennifer S Mills, Kenneth Fung, Lee Steel, Jonathan A Weiss

**Affiliations:** 1 York University, Canada; 2 Centre for Addiction and Mental Health, Canada; 3 University of Toronto, Canada

**Keywords:** acceptance and commitment therapy, autism, caregivers, interventions—psychosocial/behavioral, mental health

## Abstract

Emerging research shows that Acceptance and Commitment Therapy (ACT) may improve mental health for caregivers. Parents of autistic children, adolescents, and adults (*N* = 54) were randomly assigned to either complete a brief group-based ACT intervention or remain on the waitlist. Participants completed surveys immediately prior to randomization, and 3-, 7-, and 17-weeks post-randomization. The primary outcome was depression symptoms and secondary outcomes included stress, goal attainment, positive affect, ACT psychological processes, child mental health, and family functioning. Mixed effects linear models testing Group × Time interaction indicated the Treatment group (*n* = 27) demonstrated greater post-intervention improvements than the Waitlist group (*n* = 27) in parent depression (*p* = .03, *d* = −0.64) and family distress (*p* = .04, *d* = −0.57). Treatment group parents also reported greater short-term gains in positive affect (*p* =< .001, *d* = 0.77) and personal goal attainment (*p* = .007, *d* = 0.80), compared to the Waitlist group. Although there was no significant Group × Time interaction for other outcomes, stress (*b* = −2.58, *p* = .01), defusion (*b* = −3.78, *p* = .001), and experiential avoidance (*b* = −4.22, *p* = .01) showed improvement for the Treatment group, but not the Waitlist group, at post-intervention. All Treatment group improvements were maintained at follow-up. Results suggest that a brief ACT group intervention is efficacious for improving some aspects of mental health for parents of autistic children.

**Lay abstract:**

Parents of autistic children commonly experience difficulties with their own mental health. This study looked at the effects of a brief group-based Acceptance and Commitment Therapy program, developed for parents of autistic children, youth, and adults. ACT focuses on increasing psychological flexibility, which is the ability to be mindful and accepting of difficult thoughts and experiences, shown to be important for mental wellness. Participants included 54 parents of autistic people, ages 3–34. Parents were randomly divided into two groups: a Treatment group that received the intervention right away, and a Waitlist group that completed the program after the Treatment group completed the trial. All parents filled out questionnaires right before the program began, and at 3, 7, and 17 weeks after randomization. Compared to the group that was waiting to participate in the program, parents in the Treatment group reported greater improvements in depression and family distress, and these improvements were still present 4 months later. Parents in the Treatment group also reported short-term improvements in their positive feelings and personal goals, compared to those waiting. Results showed that ACT may help improve some aspects of mental health for parents of autistic children, but further research is recommended.

Parents of autistic children^1^ commonly experience greater levels of stress, anxiety, and depression compared to parents of children who do not have a disability, and their levels of chronic stress have been shown to surpass levels experienced by parents of children with other developmental disabilities (Bitsika & Sharpley, 2004; Estes et al., 2009). Given the high stress experienced by parents of autistic children (Hayes & Watson, 2013), it follows that parents would experience their own challenges that require parent-focused support.

Acceptance and Commitment Therapy (ACT) has emerged as an intervention with a strong evidence base supporting its use for caregivers (Han et al., 2021). The ACT framework focuses on helping individuals improve psychological flexibility, which involves increasing openness and mindful awareness in the present moment, and acceptance of difficult experiences (Hayes et al., 2006). The overall aim of ACT is not to decrease psychological distress, but to increase one’s effectiveness in pursuing values that are personally meaningful, even in the context of difficult symptoms or circumstances (Harris, 2006). In addition to rewards and positive impacts of raising an autistic child that many parents report (Kayfitz et al., 2010), parents of autistic children also describe making significant adjustments to their lives to support their child’s care, such as career changes, or devoting very limited time to social or personal activities (Meirsschaut et al., 2010). They may have difficulty navigating inaccessible care systems, unsupportive social systems, or adjusting to a parenting experience that can involve a high level of daily childcare demands (Meirsschaut et al., 2010). As a result, parents may experience difficult thoughts and feelings in response to these real challenges and stressors. Acceptance, therefore, may be a more appropriate framework than treatment modalities that focus on problem-solving. The use of psychological acceptance has also been shown to be an important protective factor in the well-being of parents of autistic children (Jones et al., 2014; Weiss et al., 2012). In addition, parents of autistic children report that they employ more avoidance coping (i.e. cognitive distancing/minimizing; escape behaviors) than parents of children without autism, which may be related to increased strain on the family system (Sivberg, 2002). Avoidance coping, although sometimes adaptive in the short term, is associated with increased stress and mental health problems over time (Hastings et al., 2005). As such, these parents may benefit from interventions that help decrease the use of psychological avoidance and promote psychological acceptance.

There is a growing literature demonstrating the effectiveness of ACT among parents of autistic children. In the first published study of ACT for parents of autistic children, Blackledge and Hayes (2006) delivered a 2-day workshop to 20 parents. Participants demonstrated post-intervention improvements in self-reported depression, distress, experiential avoidance, and cognitive fusion. In another study, two 4 h ACT workshops were delivered to five mothers of children with intellectual and developmental disabilities who displayed high levels of challenging behavior (Reid et al., 2016). Analysis of post-intervention interviews revealed that parents reported a better ability to cope with stress, increased emotional wellbeing for themselves and their child, and the ability to take a more mindful perspective on their difficulties. In a multiple baseline repeated measures analysis of three mothers of autistic children who participated in six 90-min sessions of ACT, parents increased in their use of values-guided behaviors (Gould et al., 2018) post-training and 6 months later.

In India, Poddar and colleagues (2015) delivered ACT individually to five mothers of autistic children over 10 sessions. Improvements in parent self-reports of depressive and anxious symptoms, psychological flexibility, and quality of life were noted post-intervention. In Iran, an 8-week group ACT intervention provided to 12 mothers was superior to 8 weeks of individual counseling for reducing self-reports of depression and experiential avoidance, although group assignment was not random (Joekar et al., 2016).

In a study with a slightly larger sample, Lunsky and colleagues (2018) delivered a brief ACT workshop to 29 mothers of autistic children and young adults. In addition to having ACT expertise, facilitators were also parents of autistic children. Post-intervention, improvements were seen in parents’ self-reports of depression, stress, perceived physical health, and social isolation, and improvements in all but social isolation were maintained at follow-up. A second study of this cohort reported that mothers also indicated improvements in cognitive fusion, psychological flexibility, and values-consistent action, which were maintained at follow-up (Fung et al., 2018a).

One of the only randomized controlled trials (RCTs) of ACT for parents focused on parents of children with cerebral palsy, where researchers added a 4 h ACT training for parents who were already receiving a parenting intervention, compared to a randomly assigned waitlist control group and a parent training only group. The addition of ACT training resulted in improved parent reports of child quality of life and reduced parental depression and stress, compared to the waitlist group (Whittingham et al., 2015). This study did not compare receiving only ACT to a control condition, making it difficult to determine the relative impact of ACT on the improvements observed. In the only RCT to date testing the efficacy of ACT for parents of autistic children (age 5–13), nine parents receiving Applied Behavior Analysis (ABA) participated in a 4 h ACT workshop, compared to nine parents who received ABA only (Hahs et al., 2018). The ACT group demonstrated significantly greater improvements compared to the ABA-only group in self-reports of mindfulness, experiential avoidance, cognitive fusion, consistency toward personal values, and depression, but no follow-up was employed.

ACT has yet to be evaluated in parents of autistic children using both a randomized controlled design and a large enough sample to increase generalizability to the larger population of parents of autistic children. Further, while there is evidence that in families of autistic children, parent-focused interventions can also serve to improve child behavior and mental health (Neece, 2014; Singh et al., 2006, 2014) and family adjustment (Tonge et al., 2006), no studies have examined child and family outcomes following ACT interventions for parents. Finally, as ACT is meant to improve psychological flexibility and positive functioning while not necessarily decreasing symptoms, it is essential that evaluations of ACT include outcome measures that assess positive aspects of parent functioning as well, rather than focusing only on reduction of problems.

## Aim

The current study tested the efficacy of a brief, manualized group-based ACT workshop developed for parents of children, adolescents, and adults^2^ with neurodevelopmental disabilities, including autism. A randomized controlled design compared a Treatment group that completed the program immediately to a Waitlist control condition. The primary outcome was reduction in parent depression, and secondary outcomes included other aspects of parent mental health, positive functioning, and goal attainment. We also investigated the extent to which the intervention impacted the mental health of children of participating parents, and overall family functioning. We predicted that parents who were offered the ACT program would report improvements in their mental health, positive functioning, goal attainment, and in their child’s mental health and family’s functioning, relative to the waitlist condition.

## Method

### Participants

Participants included 54 parents of autistic children, adolescents, and adults ages 3 to 34 years. To be included in the study, parents had to provide documentation of their child’s autism diagnosis from a health care practitioner qualified to diagnose autism in Ontario, Canada (i.e. clinical psychologist, pediatric/general practitioner physician, or psychiatrist). Additional inclusion criteria were the ability to speak English and identify therapy goals, and no prior exposure to ACT.

### Program description

The intervention was a three-session workshop, developed by Fung and colleagues (2018b) and described in detail by Lunsky and colleagues (2018). The first session involved a 3 h introduction to the ACT processes, and participants were introduced to mindfulness through an experiential guided imagery exercise. The second full-day session occurred the following day and included didactic, experiential, and mindfulness activities to demonstrate the concepts of ACT, and linked material to the experience of parenting an autistic person. Guided activities were interspersed with paired sharing, group discussion, and videos. At the end of session 2, participants committed to engaging in one action that aligned with their values. A 3 h refresher session was conducted 1 month later.

### Measures

#### Sample characteristics

The parent-report *Social Communication Questionnaire–Lifetime version* (SCQ; Rutter et al., 2003) was used to measure autism symptoms. The parent-report *Scales of Independent Behavior–Revised* Short Form (SIB-R; Bruininks et al., 1996) measured adaptive functioning. The SIB-R uses a standard score (*M* = 100, *SD* = 15, Range: 0–200).

#### Primary outcome

##### Depression

Symptoms of depression were measured using the 7-item Depression subscale of the *Depression Anxiety Stress Scale–21* (DASS-21; Lovibond & Lovibond, 1995). Item responses reference the past week and use a 4-point scale (0 = Never to 3 = Almost Always). Subscale scores can fall into the ranges of normal, mild, moderate, severe, or extremely severe. These severity labels characterize the range of scores in the population; a mild score does not indicate mild level of disorder and is below the typical severity of those seeking help (Lovibond & Lovibond, 1995). Therefore, a “moderate” score (7 or higher) was used to define depression of clinical concern. Internal consistency for Depression was good in the current sample (α = 0.89).

#### Secondary outcomes

##### Parent mental health and positive functioning

The 7-item Stress subscale of the DASS-21 was used to measure symptoms of stress. Internal consistency was α = 0.84 in the current sample.

Two subscales of the *Parenting Stress Index–Fourth Edition* (PSI-4; Abidin, 2012) were used. The 6-item Isolation subscale measures perceived social isolation and the 5-item Health subscale measures perceived physical health in relation to parenthood. Parents were asked to reference the past week. Items were rated on 5-point scale (1 = Strongly Agree to 5 = Strongly Disagree). The PSI-4 has been previously used with parents of autistic children (Feinberg et al., 2014; Lunsky et al., 2018; Thullen & Bonsall, 2017). In this sample, internal consistency was acceptable (Isolation α = 0.76, Health α = 0.70).

The *Positive & Negative Affect Schedule* (PANAS; Crawford & Henry, 2004) is a 20-item measure that assesses respondent’ experiences of different positive and negative emotions over the past week. Only the positive affect subscale was used, which has items that include 10 positive (i.e. active, determined) mood words, and respondents report the extent to which they experienced each mood over the last week (1 = Not at all to 5 = Extremely). The PANAS demonstrated excellent internal consistency in the current sample (α = .90 for Positive Affect) and in previous studies with parents of autistic children the Positive Affect subscale has been associated with other positive parental outcomes (e.g. optimism, psychological well-being; Ekas et al., 2010).

To assess goal attainment, at screening, participants identified three goals they hoped to attain through completing the workshop. At baseline, goals were refined with the assistance of a clinically-trained graduate student to ensure they were Specific, Measurable, Attainable, Relevant, and Timed, and *Goal Attainment Scaling*, a procedure typically used in rehabilitation intervention, was used to track progress (Bovend’Eerdt et al., 2009). For each goal, operationalized definitions were created to describe the participant’s target behaviors if their goal was achieved (0), slightly exceeded (+1), greatly exceeded (+2), not quite achieved (−1) or nowhere near (−2), to create an individualized reference guide. At each assessment time point, participants were electronically sent their guide, which they referenced to select the number that represented their progress toward each goal.

For the analysis, participants’ highest goal attainment score at each time point was used (i.e. the goal for which the participant indicated the highest achievement rating could differ between time points). We analyzed participants’ highest achieved goal (and thus the goal they were prioritizing) rather than totaling achievement of all goals, as the three goals may not have been considered equally important to participants. Focusing on one priority goal allowed for the examination of what meaningful change in one area could look like. Other research using goal attainment scaling has found utility in selecting the highest-ranking goal for analysis (Jennings et al., 2018; McGarrigle & Rockwood, 2020). To capture the effect of the program on whether goals were attained or not, the resulting values were dichotomized into 0 (*not quite achieved* or *nowhere near achieved*) and 1 (*achieved* or *slightly exceeded* or *greatly exceeded*). Past research on goal scaling has also analyzed reports in this manner (e.g. Chiarello et al., 2020; Jennings et al., 2018).

##### ACT process measures

The *Acceptance & Action Questionnaire-II* (AAQ-II; Bond et al., 2011) was used to measure psychological flexibility and acceptance. The AAQ-II is a 7-item questionnaire on which items are rated on a 7-point scale to provide an overall score. Higher scores indicate greater levels of acceptance and less experiential avoidance. The AAQ-II showed excellent internal consistency, α = .92. The AAQ and AAQ-II have been used previously in studies of parents of autistic children (Blackledge & Hayes, 2006; Hahs et al., 2018 respectively).

The *Valued Living Questionnaire* (VLQ; Wilson et al., 2010) is a two-part questionnaire that measures the extent to which respondents are in contact with their chosen values in everyday life. First, the participant indicates the importance they place on 10 life areas (e.g. friendship, work, spirituality), on a 10-point scale from Not at all important to Very important. In the second part, they indicate how consistent their actions currently are in each valued area on a 10-point scale, ranging from Completely inconsistent to Completely consistent. In this sample, the VLQ demonstrated acceptable internal consistency (Importance α = .69, Consistency α = .83, Valued Living Composite α = .76). The VLQ has been used in other studies with parents (Fung et al., 2018a).

The *Cognitive Fusion Questionnaire* (CFQ; Gillanders et al., 2014) is a 7-item measure used to assess the degree to which respondents report currently feeling distressed by, getting caught up with, or struggling to let go of thoughts. Statements are rated on a 7-point Likert-type scale (1 = Never true to 7 = Always true). Internal consistency was excellent in the current sample, α = .93. The CFQ has been used in studies of parents of autistic children (Fung et al., 2018a; Hahs et al., 2018).

The *Bangor Mindful Parenting Scale* (BMPS; Jones et al., 2014) is a 15-item questionnaire that measures parents’ degree of mindfulness in their parenting and interactions with their children. Respondents rate items on a 4-point scale (0 = Never true to 3 = Always true). It has been used previously in studies of parents of children with autism and other disabilities (Lunsky et al., 2015). Internal consistency for the total score was α = .79 in the current sample.

##### Youth mental health and family functioning

The *Strengths & Difficulties Questionnaire* (SDQ; Goodman, 2001) assesses mental health difficulties broadly in children and youth. In line with SDQ guidelines, when administered at baseline, parents were asked to reference the past 6 months, and at subsequent time points the reference period was the past 1 month. The measure has subscales for Emotional Symptoms, Conduct Problems, Hyperactivity-Inattention, Peer Problems, Prosocial Behavior, Impact, and Total Difficulties. Respondents provided item ratings on a 3-point scale (1 = Not true to 3 = Certainly true). Internal consistencies were acceptable for most subscales of the SDQ in the current sample, ranging from α = .69 to .72, except Peer Problems (α = .46). The SDQ is often used in studies of autistic children (Allik et al., 2006; Chalfant et al., 2007).

The *Brief Family Distress Scale* (BFDS; Weiss & Lunsky, 2011) was used to obtain a measure of the current level of crisis parents perceive in their family. Respondents indicate, on a scale from 1 to 10, which numbered statement most accurately represents the state of their family’s current distress. Statements range from 1 = *everything is fine, my family and I are not in crisis at all*, 5 = *things are very stressful, but we are getting by with a lot of effort*, 10 = *we are currently in crisis, and it could not get any worse*. The BFDS has been used in other studies of parents of children with developmental disabilities (Hastings, 2016; McKenzie et al., 2017).

The General Functioning subscale of the *McMaster Family Assessment Device* (FAD; Epstein et al., 1983) was used as a measure of family functioning over the past 2 weeks. Responses for each of the 12 items are provided on a 4-point scale, ranging from Strongly agree to Strongly disagree. This tool has been used in parents of children with disabilities (Brown et al., 2015) and had good internal consistency (α = .82) in the current sample.

### Procedure

#### Recruitment

**Figure 1.**
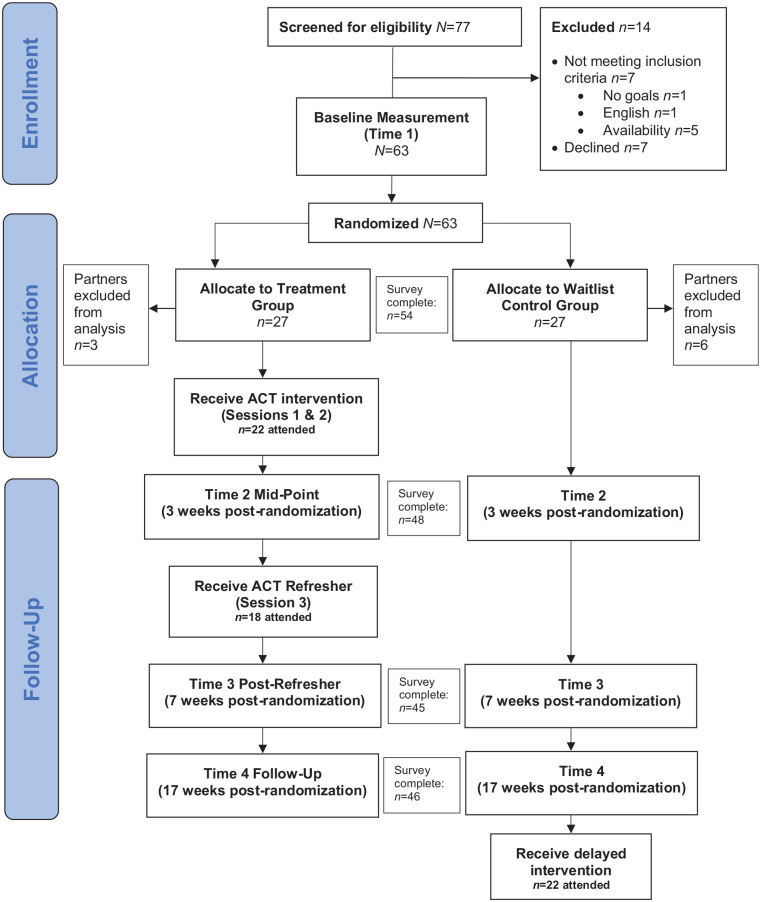
CONSORT flow diagram.

The study was approved by ethics boards at York University, the Centre for Addiction and Mental Health, and Surrey Place in Toronto, Ontario. This study was registered prior to data collection with the ISRCTN registry (97093664). See Supplemental Table 1 for the Consolidated Standards of Reporting Trials (CONSORT) Checklist. Participants were recruited from postings on websites of child autism community agencies, autism service e-newsletters, and referrals from the community between January-November 2019. Interested parents were invited to participate in a telephone screening interview to assess eligibility. For participants who met screening criteria, study procedures were explained, and written informed consent was obtained. As shown in Figure 1, 77 participants were screened, with 7 excluded for not meeting inclusion criteria, and 7 declining to participate. Power analyses using G*Power 3.1 indicated that a sample size of 55 would be sufficient to detect medium effects. No participants reported experiencing harm from participation in the trial.

#### Randomization

Following the baseline appointment, the first author randomized parents into either the Treatment or Waitlist group using online randomization software (Urbaniak & Plous, 2015). Participant randomization was stratified by gender, to ensure approximately equal numbers of males and females in each group. Sixty-three participants were randomized. If partners participated together, one was randomly excluded from the analysis (*n* = 9), leaving 27 in each group. Parents in the Treatment group participated in their assigned ACT workshop within 2 weeks. Waitlisted parents participated in the intervention after the follow-up time point.

#### Study design

Following participant screening, baseline data were collected within 2 weeks prior to the commencement of the workshop. Parents completed demographic and evaluation measures online, and goals were defined during a phone appointment. Child social communication abilities and adaptive functioning were assessed at baseline; the SCQ was administered online, and the SIB-R was completed during the phone appointment in interview format.

All outcome measures (DASS-21, PANAS, PSI-4, AAQ-II, VLQ, CFQ, BMPS, BFDS, FAD, SDQ, goal attainment) were administered online at 7 weeks post-baseline (post-refresher/ Time 3) and 17 weeks post-baseline (follow-up/Time 4). The primary outcome measure (DASS-21 Depression), as well as four additional brief measures (DASS-21 Stress, PANAS, BFDS, goal attainment) were re-administered at the mid-point of treatment (3 weeks post-randomization/Time 2). These measures were selected to obtain a brief snapshot of four areas of functioning (i.e. mental health, positive functioning, family distress, and behavior change) immediately after the first two intervention sessions/wait. Following Time 4 data collection, the Waitlist group participated in the intervention.

### Community involvement

Families of autistic individuals were involved in this study. Parents of autistic people designed the intervention (Fung et al., 2018b) and co-facilitated every workshop, working in partnership with clinicians. No autistic people were involved in the research process.

### Data analysis

Using SPSS version 27, we performed a series of linear mixed effects regression analyses at each post-treatment time point of the relationship between Group and each outcome measure (DASS-21 Depression, DASS-21 Stress, PANAS, PSI-4, AAQ-II, VLQ, CFQ, BMPS, BFDS, FAD, SDQ). As fixed effects, we entered Group (Treatment/Waitlist), Time (two levels: Baseline and Time 2 or 3), and Group × Time interaction into the model. The interaction directly tested whether Treatment and Waitlist groups showed a different pattern of change in outcome measures at the different study time points. As random effects, we included an intercept for subjects to account for repeated measurements. Mixed effects models accommodate for missing values using the maximum likelihood method, and therefore, this analysis included all randomized participants (*N* = 54 [9 partners excluded]).

Cohen’s *d* effect sizes were calculated using the difference in change scores between Treatment and Waitlist groups, divided by the pooled standard deviation at baseline. Effect size magnitudes were defined as: negligible (<0.20), small (0.20–0.49), medium (0.50–0.79), and large (⩾0.80) (Cohen, 1992).

Differences between the Treatment and Waitlist groups in their goal attainment (i.e. goal achieved/not achieved) were tested using Pearson chi-square analyses. Missing goal attainment values were imputed in SPSS using multiple imputation, including all demographic variables, baseline scores, and variables in the analysis as predictors. Chi-square values from 10 imputed datasets were pooled in R using the micombine.chisquare function from miceadds (Enders, 2010). This function produces a combined statistic, which is approximately F-distributed. Odds ratio was used as the effect size measure.

Two analyses included treatment completers only from the Treatment group (*n* = 18), who completed the full 3-day intervention with refresher, in addition to all Waitlist participants (*n* = 27). First, for depression, the number of participants in each group who moved from the clinical to non-clinical range was calculated at Time 2 and Time 3. The interaction between Group and improvement to the non-clinical range was tested using Pearson chi-square analyses. Second, to assess whether improvements were maintained at 4-month follow-up (only possible for the Treatment group), linear mixed effects regression analyses were performed on treatment completers (*n* = 18) with Time (two levels: Time 3 and Time 4) as the fixed effect, and subjects’ intercept as a random effect.

## Results

### Baseline demographics

There were no significant differences in participant characteristics at baseline (see Table 1). Participants were 83% female, two thirds had completed university, and approximately one third were currently taking psychotropic medication. All 54 participants completed the survey at Time 1. At Time 2, 48 participants completed the survey (88.9%), at Time 3, 45 participants (83.3%), and at Time 4, 46 participants (85.2%).

**Table 1.** Group differences in parent and child characteristics at baseline.

Variable	Treatment *n* = 27	Waitlist *n* *=* 27	*t* or χ^2^	*p*
Parents				
Age *M* (*SD*)	47.11 (7.66)	49.56 (9.80)	*t* = −1.02	.31
Range	36–66	32–69		
Sex *n* (*%*)			χ^2^ = 1.20	.27
Female	24 (88.9%)	21 (77.8%)		
Male	3 (11.1%)	6 (22.2%)		
Marital status *n* (*%*)			χ^2^ = 0.91	.34
Married/common law	19 (70.4%)	22 (81.5%)		
Single/divorced/separated	8 (29.6%)	5 (18.5%)		
Current sleep/psychotropic medication			χ^2^ = 4.21	.12
Yes	6 (22.2%)	12 (44.4%)		
No	20 (74.1%)	12 (44.4%)		
Missing	1 (3.7%)	3 (11.1%)		
Current individual/group/family therapy			χ^2^ = 4.60	.10
Yes	8 (29.6%)	4 (14.8%)		
No	10 (37.0%)	16 (59.3%)		
Missing	9 (33.3%)	7 (25.9%)		
Ethnicity *n* (%)			χ^2^ = 8.36	.40
Chinese	2 (7.4%)	2 (7.4%)		
East Asian	0	1 (3.7%)		
Filipino	0	1 (3.7%)		
South Asian	0	2 (7.4%)		
Black	1 (3.7%)	0		
White	20 (74.1%)	19 (70.4%)		
Latin American/Hispanic	1 (3.7%)	0		
Other/Prefer not to answer	3 (11.1%)	2 (7.4%)		
Education *n* (%*)*			χ^2^ = 1.89	.26
High school graduate	1 (3.7%)	1 (3.7%)		
Graduated college	6 (22.2%)	5 (18.5%)		
Some university	2 (7.4%)	3 (11.1%)		
Graduated university	10 (37.0%)	7 (25.9%)		
Post-graduate degree	8 (29.6%)	11 (40.7%)		
Household income (CAD) *n* (*%*)			χ^2^ = 3.18	.53
<$50,000	2 (7.4%)	2 (7.4%)		
$50,000–US$99,999	8 (29.6%)	6 (22.2%)		
$100,000–US$149,999	5 (18.5%)	5 (18.5%)		
⩾$150,000	10 (37.0%)	8 (29.6%)		
Prefer not to answer	2 (7.4%)	6 (22.2%)		
Children				
Age *M* (*SD*)	15.07 (7.92)	14.07 (7.77)	*t* = 0.47	.64
* Range*	3–34	3–27		
Sex *n* (%)			χ^2^ = 1.03	.31
Female	7 (25.9%)	4 (14.8%)		
Male	20 (74.1%)	23 (85.2%)		
Years since autism diagnosis *M* (*SD*)	7.26 (7.80)	8.35 (7.11)	*t* = −0.47	.64
* Range*	0–33	1–22		
Ethnicity *n* (%)			χ^2^ = 5.02	.66
Indigenous	0	1 (3.7%)		
Chinese	1 (3.7%)	1 (3.7%)		
South Asian	0	2 (7.4%)		
Black	1 (3.7%)	0		
White	22 (81.5%)	21 (77.8%)		
Other/prefer not to answer	3 (11.1%)	2 (7.4%)		
SIB-R Standard Score *M* (*SD*)	75.63 (27.75)	63.41 (36.64)	*t* = 1.38	.17
* Range*	12–118	0–124		
SCQ Total Score *M* (*SD*)	18.56 (6.81)	19.78 (5.73)	*t* = −0.71	.48
* Range*	6–32	11–29		

*SD*: standard deviation; *SIB-R*: Scales of Independent Behavior–Revised; *SCQ*: Social Communication Questionnaire.

### Primary outcome

#### Depression

At Time 2 (mid-point), following the main 2-day workshop, parents in the Treatment group demonstrated significant reductions in depression scores (*b* = −3.21, *p* = .001), whereas these improvements were not seen for the Waitlist group (*b* = 0.46, *p* = .51), with a large Time × Condition interaction (see Table 2). At Time 3 (post-refresher), the Treatment group showed sustained reductions in depression scores (*b* = −2.60, *p* = .03) and the Waitlist continued to show no reduction in depression (*b* = 0.08 *p* = .88), with a medium Time × Condition effect.

**Table 2.** Primary outcome for treatment and waitlist groups from baseline using linear mixed effects model.

Measure	EMM (*SE*)		Time × Condition Effect	*p*	*d*
	Treatment	Waitlist			
*DASS-21* Depression					
Baseline	7.12 (0.95)	5.48 (0.57)			
Time 2	3.91 (0.57)	5.95 (0.87)	*F*(1, 48.31) = 10.53	.002	–0.87
Time 3	4.53 (0.97)	5.56 (0.82)	*F*(1, 48.08) = 5.02	.03	–0.64

*SE*: standard error; DASS-21: Depression Anxiety Stress Scale; EMM: estimated marginal mean.

In the Treatment group, 18 participants completed the full 3-day intervention, including the refresher. Improvements observed in the Treatment group were maintained at 4-month follow-up, with no differences among treatment completers between Time 3 and Time 4 depression scores (*b* *=* −0.14; *p* = .90; see Supplemental Table 3 for Time 4 analyses).

##### Improvement from clinical to non-clinical range

Out of 18 treatment completers, 12 had depression scores in the moderate or higher range at baseline. Of the 12, 10 parents entered the non-clinical range for depression by Time 2 (83%), and 8 remained non-clinical by Time 3 (67% improvement). In the Waitlist group, none of the 10 participants who were in the clinical range at baseline moved to the non-clinical range at Time 2, and 3 became non-clinical by Time 3 (30%). At Time 2, there was a significant interaction between Group and moving to the non-clinical range (χ^2^ (1) = 15.28, *p* =< .001), which attenuated by Time 3 (χ^2^ (1) = 2.93, *p* = .09). By Time 3, two Waitlist participants originally in the non-clinical range moved to a clinical score on the depression subscale, as did one Treatment group participant.

### Secondary outcomes

#### Parent mental health and positive functioning

Values for between-group analyses for all secondary outcomes can be found in Supplemental Table 2. There was no significant Time × Condition effect for stress at Time 2 or Time 3. At Time 2, while the Treatment group did not have a significant reduction in stress from baseline, there was a trend toward reduction (*b* = −1.67, *p* = .07), which was not observed in the Waitlist group (*b* = 0.22, *p* = .80). At Time 3, significant improvements in stress from baseline were observed in the Treatment group (*b* = −2.58, *p* = .01), but not for the Waitlist group (*b* = −0.99, *p* = .17). At follow-up, maintenance of the improvements in stress between baseline and Time 3 was seen, with no significant changes from Time 3 to Time 4 for treatment completers (*b* = 0.67, *p* = .43).

At Time 2, there was a significant Time × Condition interaction for positive affect, with a small effect size. The Treatment group showed an increase in positive affect (*b* = 2.76, *p* = .02), whereas the Waitlist group had lower positive affect at Time 2 (*b* = −2.95, *p* =< .001). At Time 3, there was no treatment effect observed for positive affect.

At Time 3, there was no significant Time × Condition interaction for parenting stress-related health, but there was a trend toward more improvement in the Treatment group (*b* = −0.89, *p* = .16) than the Waitlist group (*b* = 0.84, *p* = .20). For isolation, there was no Time × Condition effect, nor any within-group change for either group.

##### Goal attainment

At Time 2, 81.9% of the Treatment group had met at least one of their goals, compared to 43.0% of the Waitlist group, and there was a significant association between Group and goal attainment, *F*(1, 461.41) = 7.47, *p* = .007, *d* = 0.80. Based on the odds ratio, the odds of achieving or exceeding one’s goal was 5.99 times higher if in the Treatment group than in the Waitlist group (95% confidence interval:1.73, 20.70). This association was not present at Time 3, *F*(1, 394.16) = 1.45, *p* = .23, *d* = 0.33 or at Time 4, *F*(1, 637.51) = 0.12, *p* = .73, *d* = 0.09. At Time 3, 75.2% of the Treatment group was achieving or exceeding their goal, compared to 58.1% for the Waitlist group.

#### ACT process measures

There was no Time × Condition effect for any ACT process measures. Looking at within-group change for each group, improvements were observed in the Treatment group for experiential avoidance (*b* = −4.22, *p* = .01) and cognitive fusion (*b* = −3.78, *p* = .001), whereas none were observed for the Waitlist group (*b* = −0.86, *p* = .56 and *b* = −2.03, *p* = .11 respectively). There was no significant within-group change for valued living or mindful parenting at Time 3 for either group. At 4-month follow-up, maintenance of the Treatment group’s improvements between baseline and Time 3 was seen for experiential avoidance. While not significant, there was also a trend toward further improvement (*b* = −2.76; *p* = .07). There was a change observed in cognitive fusion, indicating further improvements since Time 3 for the Treatment group (*b* = −2.44, *p* = .04).

#### Family functioning and youth mental health

Between baseline and Time 2, being in the Treatment group was associated with reductions in family distress (*b* = −1.09, *p* =< .001), not seen for the Waitlist group (*b* = −0.31, *p* = .13). The Time × Condition effect was significant with a small effect size. At Time 3, a medium Time × Condition effect was seen for family distress, with parents in the Treatment group (*b* = −1.00, *p* = .001) demonstrating greater improvement compared to the Waitlist group (*b* = −0.10, *p* = .77). At 4-month follow-up, maintenance of the improvements in family distress between baseline and Time 3 was seen, with no significant changes from Time 3 to Time 4 for treatment completers (*b* = 0.05; *p* = .77). There was no within-group change or Time × Condition effect for general family functioning or youth mental health.

## Discussion

The current study used an RCT design to test the efficacy of a brief, group-based ACT intervention for mothers and fathers of autistic children, adolescents, and adults. Overall, the intervention resulted in gains for parents, with the greatest treatment effects observed for parent depression and family distress, which were maintained at the 4-month follow-up. Parents also reported short-term gains with respect to personal goal attainment, and small changes in positive affect. Although there was no significant Group × Time interaction for stress, cognitive fusion, and experiential avoidance, all these outcomes showed improvement, with small effect sizes, for the Treatment group at post-intervention, while the Waitlist group did not improve; these within-group changes were also maintained at follow-up. Parents did not report any significant improvements with respect to mindful parenting, valued living, overall family functioning, or child mental health.

### Parent mental health and positive functioning

The most consistent treatment effect was seen for parent depression. These findings align with previous studies of ACT for parents of autistic people (Blackledge & Hayes, 2006; Joekar et al., 2016; Lunsky et al., 2018; Poddar et al., 2015), wherein parent self-reports of depression decreased post-intervention. In our study, 67% of Treatment condition participants with clinical levels of depression were in the normal range at Time 3, compared to only 30% of those in the Waitlist condition. While Blackledge and Hayes had some evidence for cognitive defusion as a mediator for depression improvements, and studies of ACT in other populations also show post-treatment defusion mediating changes in depression (Zettle et al., 2011), our sample showed less change in defusion than found in prior research. Fung and colleagues (2018) did report that post-ACT intervention improvements in mental health for mothers of autistic children were mediated by improvements in values-consistent action. Although our sample did not show change in their reports of how consistently they acted in accordance with their values, they did in action toward individually-set goals, which could underlie the changes in depression observed. Additional research with a larger cohort of participants would help to better elucidate the relationship between valued living, goals, cognitive fusion, and depression in parents of autistic youth.

Conversely, no significant differences between groups for stress were reported. Parents of autistic children can experience chronic stressors, including high levels of mental health problems in their autistic youth (e.g. Simonoff et al., 2008). Indeed, in the current study, the average child mental health problems score was in the “very high” range, including specific conduct problems ranging from “raised” to “high,” and the overall impact of these problems in the “very high” range. These family characteristics may require targeted interventions before pronounced differences are seen in parents’ personal experience of stress.

Parents who participated in ACT had greater increases in positive affect compared to the Waitlist group at Time 2, though the effect was small and dissipated by Time 3. The link between positive affect and perceived social support in parents of children with disabilities has been repeatedly demonstrated (Findler et al., 2016; Smith et al., 2012), and it follows that positive affect would increase immediately after an intensive workshop designed to allow parents with similar experiences to connect. This may not have been maintained as time passed, without sustained social connections or ongoing engagement with the material.

Goal attainment, similarly, showed a large group difference at Time 2, which was no longer apparent at Time 3. At Time 2, the Treatment group was six times more likely to report achieving or exceeding one of their goals, with 82% of the Treatment parents and only 43% of the Waitlist parents indicating goal achievement. Recent research on goal attainment indicates that ongoing progress is most likely when goals are set just slightly out of reach, balancing challenge while preventing discouragement (Chevance et al., 2021). Further, ACT encourages parents to shift their behavior to align with their most closely held values. Since many Treatment group participants had already achieved or exceeded their highest-rated goal just after the workshop weekend, they might have benefited from re-setting their goals higher after reaching them, or based on values they identified and prioritized in the workshop. Last, as the highest-rated goal may have differed at different time points, we cannot say whether attainment of a particular goal was maintained for each participant at subsequent measurement times, only that many reported attaining at least one of their goals.

### ACT psychological processes

In terms of the four ACT psychological processes, cognitive fusion and experiential avoidance showed small improvements in the Treatment group only, though not to a degree that resulted in a Group × Time interaction, and there was no change in mindful parenting or valued living. As shown in other studies of ACT interventions, some process-related changes take several months to become evident, particularly with respect to behavior change (González-Menéndez et al., 2014; Kohtala et al., 2017). Further, for mindfulness-based interventions, there is a strong relation between degree of mindfulness practice at home and amount of improvement in mindfulness and wellbeing (Carmody & Baer, 2009; Greenberg et al., 2018; Parsons et al., 2017). Published ACT manuals emphasize the importance of home practice (Hayes, 2005), and it is generally established that practice is beneficial to maximize effects of psychotherapy in general (Hoet et al., 2018). A weekly format with regular homework check-ins, or individual support, might support more practice and therefore greater change in behavior.

### Family functioning and child mental health

More distal outcomes of family functioning and child mental health were also assessed. There were strong consistent treatment effects for family distress (BFDS). Although the intervention is not intended to attenuate family crises, participants in ACT are encouraged to change their relationship to their internal and external experiences. The BFDS asks parents to provide a broad appraisal of how difficult their current situation is and post-intervention, parents might have appraised their circumstances in a different light. It is also possible that reductions in distress occurred following experiences that were not sufficiently captured in our process measures. For instance, participants engaged in positive, supportive, and relaxing activities, possibly providing a sense of greater ability to cope with stressors or a temporary sense of relief, translating into shifts in their experience of distress. Further, through engaging with fellow group members in similar situations, and sharing experiences of distress, it is possible that participants received important informal social support that helped them to manage the stressors in their lives. Results do not support the hypothesis that a brief ACT workshop would result in changes in more general family functioning and child mental health. The factors affecting mental health problems are complex and multifaceted, with many unrelated to parenting behavior or parent mental health, and require more direct management to result in improvement (White et al., 2018).

### Strengths and limitations

This study had many strengths. This was one of only two RCTs testing the efficacy of ACT for improving well-being in parents of autistic children, allowing us to elucidate the treatment effects of ACT while controlling for time and repeated measurements. In addition, the inclusion of a follow-up assessment ensures that longer-term program effects are captured. The heterogeneity of the sample, in terms of including fathers, as well as parents of autistic children spanning a range of ages, socio-communication functioning, and adaptive skills, is also a strength, and improves generalizability of findings to more parents of autistic people.

There are also key limitations to our study. Participants were recruited mainly through autism service agencies, and many were already receiving supports and services for their child, which may limit generalization to families who do not have any support, or who are unwilling to potentially wait 4 months to receive the program. Twenty-two Treatment group participants completed the main intervention weekend, and four were unable to attend the refresher one month later, leaving only 18 full “treatment completers.” In addition, all outcomes were assessed through subjective parent report, which could potentially influence the validity of results or limit some effects of the intervention being captured. Further, the high average level of household income and parent education, limited racial/ethnic diversity, and relatively low levels of parent mental health problems in this cohort limit the conclusions that can be drawn regarding the effect of ACT for parents with less education, with varying cultural backgrounds, or with greater need.

## Conclusion

Given the higher levels of distress experienced by parents of autistic individuals compared to parents who do not have a child with a disability (Keenan et al., 2016), it is crucial to identify brief, effective and accessible supports. This study aligns with the growing body of research that suggests that ACT can benefit parents across multiple domains. Important next steps are to understand more about parents’ experiences in the workshop to learn which aspects of the intervention are most useful. This might include a measure of social connection in the group. Future trials might also compare different modes of intervention delivery (i.e. weekly vs brief intensive format, in-person vs virtual delivery) as well as the effect of practice or longer-term support, on outcomes. In addition, studies may consider implementing assessments of children and families from a secondary reporter, such as a teacher or other parent, or youth self-report. Finally, it would be important to include a longer follow-up time point in additional RCTs of ACT.

## Supplemental Material

sj-docx-1-aut-10.1177_13623613231172241 – Supplemental material for Parent, child, and family outcomes following Acceptance And Commitment Therapy for parents of autistic children: A randomized controlled trial Click here for additional data file. Supplemental material, sj-docx-1-aut-10.1177_13623613231172241 for Parent, child, and family outcomes following Acceptance And Commitment Therapy for parents of autistic children: A randomized controlled trial by Andrea L Maughan, Yona Lunsky, Johanna Lake, Jennifer S Mills, Kenneth Fung, Lee Steel and Jonathan A Weiss in Autism

sj-docx-2-aut-10.1177_13623613231172241 – Supplemental material for Parent, child, and family outcomes following Acceptance And Commitment Therapy for parents of autistic children: A randomized controlled trial Click here for additional data file. Supplemental material, sj-docx-2-aut-10.1177_13623613231172241 for Parent, child, and family outcomes following Acceptance And Commitment Therapy for parents of autistic children: A randomized controlled trial by Andrea L Maughan, Yona Lunsky, Johanna Lake, Jennifer S Mills, Kenneth Fung, Lee Steel and Jonathan A Weiss in Autism

sj-docx-3-aut-10.1177_13623613231172241 – Supplemental material for Parent, child, and family outcomes following Acceptance And Commitment Therapy for parents of autistic children: A randomized controlled trial Click here for additional data file. Supplemental material, sj-docx-3-aut-10.1177_13623613231172241 for Parent, child, and family outcomes following Acceptance And Commitment Therapy for parents of autistic children: A randomized controlled trial by Andrea L Maughan, Yona Lunsky, Johanna Lake, Jennifer S Mills, Kenneth Fung, Lee Steel and Jonathan A Weiss in Autism

## Notes

1. We recognize that there are many viewpoints with respect to selecting language to describe autism and other disabilities. We use identity-first language to reflect the preference of many autistic adults and their family members (Botha et al., 2021; Kenny et al., 2016).

2. The term *children* will subsequently be used to refer to participating parents’ autistic children, regardless of the age of the child, adolescent, or adult.
