# Supplementary material for: Parent, child, and family outcomes following Acceptance And Commitment Therapy for parents of autistic children: A randomized controlled trial
Source: Autism. 2023 May 11;28(2):367–80. doi: 10.1177/13623613231172241 (PMC10851654; doi:10.1177/13623613231172241)
Supplement: sj-docx-2-aut-10.1177_13623613231172241 – Supplemental material for Parent, child, and family outcomes following Acceptance And Commitment Therapy for parents of autistic children: A randomized controlled trial [file sj-docx-2-aut-10.1177_13623613231172241.docx]

**Supplemental Table 2**

*Secondary Outcomes for Treatment and Waitlist Groups from Baseline Using Linear Mixed Effects Model*

| Measure | EMM (SE) | | Time x Condition Effect | *p* | *d* |
| --- | --- | --- | --- | --- | --- |
|  | Treatment | Waitlist |  |  |  |
| DASS-21 Stress |  |  |  |  |  |
| Baseline | 9.26 (0.81) | 8.35 (0.76) |  |  |  |
| Time 2 | 7.59 (0.83) | 8.57 (0.89) | *F* (1, 50.49) = 2.32 | .13 | -0.46 |
| Time 3 | 6.68 (0.93) | 7.36 (0.72) | *F* (1, 50.89) = 2.00 | .16 | -0.38 |
| PANAS |  |  |  |  |  |
| Baseline | 27.59 (1.51) | 29.30 (1.31) |  |  |  |
| Time 2 | 30.35 (1.41) | 26.34 (1.37) | *F* (1, 50.10) = 18.38 | **<.001** | 0.77 |
| Time 3 | 29.47 (2.00) | 28.64 (1.43) | *F* (1, 45.57) = 1.70 | .20 | 0.34 |
| BFDS |  |  |  |  |  |
| Baseline | 5.00 (0.37) | 4.82 (0.22) |  |  |  |
| Time 2 | 3.91 (0.31) | 4.51 (0.30) | *F* (1, 49.12) = 5.88 | **.02** | -0.49 |
| Time 3 | 4.00 (0.33) | 4.72 (0.38) | *F* (1, 46.31) = 4.29 | **.04** | -0.57 |
| CFQ |  |  |  |  |  |
| Baseline | 27.41 (1.70) | 27.37 (1.39) |  |  |  |
| Time 3 | 23.63 (1.57) | 25.34 (1.47) | *F* (1, 49.97) = 1.13 | .29 | -0.22 |
| AAQ-II |  |  |  |  |  |
| Baseline | 22.70 (1.74) | 21.04 (1.34) |  |  |  |
| Time 3 | 18.48 (1.68) | 20.18 (1.40) | *F* (1, 50.43) = 2.50 | .12 | -0.41 |
| BMPS |  |  |  |  |  |
| Baseline | 25.11 (1.05) | 26.70 (0.96) |  |  |  |
| Time 3 | 26.72 (1.07) | 26.38 (0.84) | *F* (1, 48.18) = 1.30 | .26 | 0.28 |
| VLQ Composite |  |  |  |  |  |
| Baseline | 44.07 (2.95) | 48.07 (2.58) |  |  |  |
| Time 3 | 46.20 (3.19) | 46.26 (3.58) | *F* (1, 46.75) = 1.13 | .29 | 0.27 |
| PSI-4 Health |  |  |  |  |  |
| Baseline | 15.52 (0.78) | 14.74 (0.77) |  |  |  |
| Time 3 | 14.63 (0.74) | 15.58 (0.90) | *F* (1, 47.45) = 3.73 | .06^+^ | -0.42 |
| PSI-4 Isolation |  |  |  |  |  |
| Baseline | 17.93 (0.85) | 18.33 (0.34) |  |  |  |
| Time 3 | 16.67 (0.76) | 18.50 (0.91) | *F* (1, 49.50) = 1.30 | .26 | -0.32 |
| FAD |  |  |  |  |  |
| Baseline | 27.89 (0.93) | 26.41 (0.88) |  |  |  |
| Time 3 | 26.57 (1.26) | 26.72 (0.91) | *F* (1, 46.73) = 1.43 | .24 | -0.34 |
| SDQ Impact |  |  |  |  |  |
| Baseline | 4.85 (0.52) | 4.37 (0.38) |  |  |  |
| Time 3 | 4.95 (0.52) | 4.06 (0.49) | *F* (1, 46.26) = 0.25 | .62 | 0.17 |

*DASS-21* Depression Anxiety Stress Scale*; PANAS* Positive and Negative Affect Schedule; *BFDS* Brief Family Distress Scale; *CFQ* Cognitive Fusion Questionnaire; *AAQ-II* Acceptance & Action Questionnaire; *VLQ* Valued Living Questionnaire; *BMPS* Bangor Mindful Parenting Scale; *PSI-4* Parenting Stress Inventory; *FAD* McMaster Family Assessment Device; *SDQ* Strengths and Difficulties Questionnaire.
