# Supplementary material for: Parent, child, and family outcomes following Acceptance And Commitment Therapy for parents of autistic children: A randomized controlled trial
Source: Autism. 2023 May 11;28(2):367–80. doi: 10.1177/13623613231172241 (PMC10851654; doi:10.1177/13623613231172241)
Supplement: sj-docx-3-aut-10.1177_13623613231172241 – Supplemental material for Parent, child, and family outcomes following Acceptance And Commitment Therapy for parents of autistic children: A randomized controlled trial [file sj-docx-3-aut-10.1177_13623613231172241.docx]

**Supplemental Table 3**

*Follow-up Change for Treatment Completers from Time 3 to Time 4 Using Linear Mixed Effects Model*

| Measure | EMM (SE) | | Time Effect | *Effect from Baseline b (SE)* | *p* |
| --- | --- | --- | --- | --- | --- |
|  | Time 3 | Time 4 |  |  |  |
| DASS-21 Depression | 5.19 (1.11) | 5.06 (0.74) | *F* (1, 18) = 0.02, *d* = -0.03 | -0.14 (1.06) | .90 |
| DASS-21 Stress | 7.00 (1.09) | 7.67 (0.95) | *F* (1, 18) = 0.66, *d* = -0.17 | 0.67 (0.82) | .43 |
| PANAS | 27.63 (2.00) | 29.06 (1.83) | *F* (1, 17.09) = 1.19, *d* = 0.17 | 1.43 (1.31) | .29 |
| BFDS | 4.45 (0.38) | 4.50 (0.45) | *F* (1, 17.94) = 0.09, *d* = 0.03 | 0.05 (0.17) | .77 |
| CFQ | 25.67 (1.81) | 23.22 (1.60) | *F* (1, 18) = 5.20, *d* = -0.33 | -2.44 (1.07) | **.04** |
| AAQ-II | 20.20 (1.90) | 17.44 (1.61) | *F* (1, 17.15) = 3.65, *d* = -0.35 | -2.76 (1.44) | .07^+^ |
| BMPS | 26.19 (1.30) | 27.61 (1.29) | *F* (1, 17.31) = 2.19, *d* = 0.26 | 1.42 (0.96) | .16 |
| PSI-4 Health | 14.83 (0.73) | 14.94 (0.86) | *F* (1, 18) = 0.03, *d* = 0.03 | 0.11 (0.70) | .88 |
| PSI-4 Isolation | 16.00 (0.65) | 16.10 (0.77) | *F* (1, 18) = 0.02, *d* = 0.02 | 0.10 (0.71) | .89 |
| FAD | 27.73 (1.51) | 27.94 (1.47) | *F* (1, 17.06) = 0.07, *d* = 0.05 | 0.21 (0.78) | .79 |
| SDQ Impact | 4.92 (0.52) | 4.67 (0.65) | *F* (1, 18) = 0.28, *d* = -0.09 | -0.25 (0.48) | .60 |
| VLQ Composite | 43.88 (3.39) | 45.24 (3.88) | *F* (1, 17.31) = 0.21, *d* = 0.09 | 1.36 (2.96) | .65 |

*DASS-21* Depression Anxiety Stress Scale; *PANAS* Positive and Negative Affect Schedule; *BFDS* Brief Family Distress Scale; *CFQ* Cognitive Fusion Questionnaire; *AAQ-II* Acceptance & Action Questionnaire; *VLQ* Valued Living Questionnaire; *BMPS* Bangor Mindful Parenting Scale; *PSI-4* Parenting Stress Inventory; *FAD* McMaster Family Assessment Device; *SDQ* Strengths and Difficulties Questionnaire.
